# Increased Medial Prefrontal Cortex and Decreased Zygomaticus Activation in Response to Disliked Smiles Suggest Top-Down Inhibition of Facial Mimicry

**DOI:** 10.3389/fpsyg.2019.01715

**Published:** 2019-07-26

**Authors:** Sebastian Korb, Robin Goldman, Richard J. Davidson, Paula M. Niedenthal

**Affiliations:** ^1^Department of Applied Psychology: Health, Development, Enhancement and Intervention, Faculty of Psychology, University of Vienna, Vienna, Austria; ^2^Department of Psychology, University of Wisconsin–Madison, Madison, WI, United States; ^3^Center for Healthy Minds, University of Wisconsin–Madison, Madison, WI, United States

**Keywords:** facial mimicry, inhibition, reward, electromyography, fMRI, medial prefrontal cortex

## Abstract

Spontaneous facial mimicry is modulated by many factors, and often needs to be suppressed to comply with social norms. The neural basis for the inhibition of facial mimicry was investigated in a combined functional magnetic resonance imaging and electromyography study in 39 healthy participants. In an operant conditioning paradigm, face identities were associated with reward or punishment and were later shown expressing dynamic smiles and anger expressions. Face identities previously associated with punishment, compared to reward, were disliked by participants overall, and their smiles generated less mimicry. Consistent with previous research on the inhibition of finger/hand movements, the medial prefrontal cortex (mPFC) was activated when previous conditioning was incongruent with the valence of the expression. On such trials there was also greater functional connectivity of the mPFC with insula and premotor cortex as tested with psychophysiological interaction, suggesting inhibition of areas associated with the production of facial mimicry and the processing of facial feedback. The findings suggest that the mPFC supports the inhibition of facial mimicry, and support the claim of theories of embodied cognition that facial mimicry constitutes a spontaneous low-level motor imitation.

## Introduction

People have the tendency to spontaneously imitate others’ actions, postures, and facial expressions (Brass et al., 2000; Heyes, 2011; Wood et al., 2016). For the face, this phenomenon is termed *facial mimicry*.

According to theories of embodied cognition, facial mimicry is a low-level motor imitation that occurs spontaneously and can facilitate and/or speed up the recognition of the observed expression through afferent feedback to the brain (Niedenthal, 2007; Barsalou, 2008; Wood et al., 2016). This view is sometimes called the “matched motor” hypothesis (Hess and Fischer, 2013). Facial mimicry can occur without conscious perception of the stimulus face (Dimberg et al., 2000; Mathersul et al., 2013; but see Korb et al., 2017), and is difficult to suppress voluntarily (Dimberg et al., 2002; Korb et al., 2010). Mimicry influences judgments of expression authenticity (Hess and Blairy, 2001; Korb et al., 2014), and the blocking of facial mimicry compromises the processing of facial expressions by reducing recognition speed and accuracy as measured with behavior (Niedenthal et al., 2001; Oberman et al., 2007; Stel and van Knippenberg, 2008; Maringer et al., 2011; Rychlowska et al., 2014; Baumeister et al., 2016), and electroencephalography (EEG; Davis et al., 2017), as well as reducing activation in the brain’s emotion centers, such as the amygdala (Hennenlotter et al., 2009).

Although of automatic origin, facial mimicry can be increased or decreased in a top-down fashion. Indeed, facial mimicry has been found to be modulated by several factors including the expresser–observer relationship (Seibt et al., 2015; Kraaijenvanger et al., 2017). For example, mimicry of happiness is reduced for faces associated with losing money, compared to faces associated with winning money (Sims et al., 2012). Such findings suggest that people spontaneously generate facial mimicry, which is inhibited or increased, depending on whether they like or dislike the person they are interacting with. Related behavioral work demonstrated that the automatic tendency to imitate seen hand gestures (thumbs up and middle finger) is modulated by their pro-social or anti-social meaning (Cracco et al., 2018). Consistent with this, in a delayed cued counter-imitative task, activation of the primary motor cortex is greater first according to a stimulus-congruent “mirror” response, and later reflecting a rule-based “nonmirror” response (Ubaldi et al., 2015).

Research employing functional magnetic resonance imaging (fMRI) and transcranial magnetic stimulation (TMS) has suggested that facial mimicry originates in lateral and medial cortical, as well as subcortical motor, premotor, and somatosensory areas (van der Gaag et al., 2007; Schilbach et al., 2008; Keysers et al., 2010; Likowski et al., 2012; Korb et al., 2015b; Paracampo et al., 2016). But which neural circuits are necessary to inhibit facial mimicry in a top-down manner? We aimed to address this question in the present study.

Several lines of research suggest that the modulation of facial mimicry may originate in the medial prefrontal cortex (mPFC). First, areas of the PFC are involved in cognitive control and emotion regulation (Miller, 2000; Ochsner and Gross, 2005; Korb et al., 2015a), and patients with prefrontal lesions often lack inhibitory control of prepotent response tendencies, such as action imitation, and can even display over-imitation of words (echolalia) and actions (echopraxia) (Lhermitte et al., 1986; De Renzi et al., 1996; Brass et al., 2003). Second, the mPFC is involved in suppressing motor imitation of hand actions, as shown by Brass et al. (2001, 2005) using a simple task, in which participants executed predefined finger movements while observing congruent or incongruent finger movements. In this task, increased activation is observed on incongruent compared to congruent trials in a network encompassing the mPFC and the temporo-parietal junction (TPJ). In this network, the right TPJ and the neighboring supramarginal gyrus allow participants to differentiate between their own and the observed movements (Silani et al., 2013), and the mPFC likely underlies the inhibition of spontaneously arising imitation tendencies. For example, Wang et al. (2011) and Wang and Hamilton (2012) found the mPFC to be a key structure for the inhibition of finger and hand movement imitation, and the integration of eye contact. Based on Wang and Hamilton (2012)’s findings, the social top-down response modulation (STORM) model specifically proposes that imitation and mimicry are top-down modulated by the mPFC in order to increase the person’s social advantage, e.g., by making her being liked more by other people who unconsciously pick up being mimicked. Similarly, in a review paper focusing on the modulation of facial mimicry by neuroendocrine factors, Kraaijenvanger et al. (2017) attribute to prefrontal cortices, including the mPFC, a central role for the processing and mimicry of emotional faces in context. However, most previous brain imaging research has focused on the imitation of hand and finger gestures; no study so far has investigated the neural correlates of the inhibition of facial mimicry.

In the current experiment we investigated the role of the mPFC in the inhibition of facial mimicry of smiles. Smile mimicry was induced using dynamic stimuli showing facial expressions of happiness and verified with facial electromyography (EMG) of the zygomaticus major (ZM) muscle, while brain activity was sampled using fMRI. The expected inhibition of facial mimicry was induced by first conditioning participants to associate specific identities with winning or losing money. Although this task does not require the inhibition of facial mimicry *per se* – as in the face version of the finger-tapping task developed by Brass et al. (2001, 2005; Korb et al., 2010) – it was used to prevent the occurrence of stimulus-locked head movements, which are likely to occur during the production of voluntary facial expressions, and which would have jeopardized the quality of the fMRI recordings. Based on prior research using the same paradigm and stimuli (Sims et al., 2012; but see also Hofman et al., 2012), we predicted greater ZM activation for happy faces associated with winning (congruent condition) than with losing money (incongruent condition). In light of previous research investigating the neural correlates of the inhibition of finger or hand movements (Brass et al., 2005), we also expected the mPFC to inhibit facial mimicry, and thus to be more activated for happy faces associated with losing than with winning money. Finally, psychophysiological interaction (PPI) analyses were used to establish the functional connectivity of the mPFC with the rest of the brain.

## Materials and Methods

### Participants

Thirty-nine right-handed, fluent English speaking, female participants were recruited via flyers posted on campus. Inclusion criteria were right-handedness, normal or corrected vision, absence of a diagnosis of psychiatric conditions, no personal history of seizures or a family history of hereditary epilepsy, and no consumption of prescribed psychotropic medication. Moreover, participants were screened with an MRI safety questionnaire. Data from three participants were excluded from analyses due to excessive head movement (over 2 mm on any of the three axes), and one participant was excluded because she misunderstood instructions and responded on every trial during the fMRI task. The final sample included in the analyses was 35 participants with a mean age of 22.9 years (*SD* = 5.1).

### Stimuli

**FIGURE 1:**
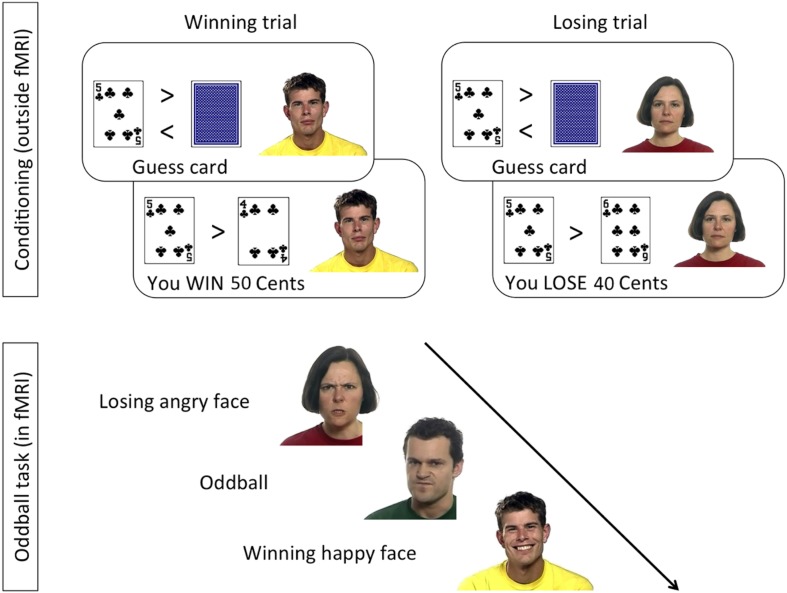
Design of the conditioning and oddball tasks, following the procedure by Chakrabarti and colleagues (Sims et al., 2012, 2014).

Stimuli were taken from the “Mindreading set” (Baron-Cohen et al., 2004^1^), as described in Sims et al. (2014). In the conditioning phase, static pictures of three faces with neutral facial expression were shown next to two playing cards (Figure 1). In the oddball task, completed inside the MR scanner, 4-s long video clips showing dynamic emotional facial expressions of anger and happiness were shown. Faces used in the oddball task included those used in the conditioning phase as well as novel (previously unseen) faces.

### Procedure

All participants signed informed consent. The study was approved by the Health Sciences IRB of the University of Wisconsin–Madison (FWA00005399). The procedure was modeled after a paradigm developed by Sims et al. (2014). All tasks were programmed and displayed with E-Prime 2.0 (Psychology Software Tools, Sharpsburg, PA, United States). Participants completed the conditioning and rating tasks in a behavioral testing room, had EMG electrodes attached, and were moved into the scanner, where they completed the oddball task.

In the conditioning task (Figure 1, top), a card-guessing game was used to condition one face identity with the experience of winning money, another identity with losing money, and two more identities with neither winning nor losing money. Contingencies were counterbalanced across participants. Specifically, on each trial a task-irrelevant face with a neutral expression was displayed next to task-relevant playing cards. To make the presence of the faces plausible, participants were told that the faces would be part of a later memory game. Participants were seated 60 cm from a 17-inch computer screen.

A blank screen initiated a trial and was shown for 250 ms, followed by the presentation of an open card, next to a neutral face. After 1.5 s a closed card appeared next to the open one. Participants’ pressed the “m” or “n” buttons (counterbalanced across participants) on the keyboard to indicate if they guessed the closed card to be higher or lower than the open card. The card then turned face up, and the outcome of the trial was indicated in the lower part of the screen. The outcome, which was displayed for 4 s, could be “You Win (+50 Cents),” “You Lose (−40 Cents),” or “Draw.” The outcome of all trials was programmed in advance, and adaptively showed a higher or lower card depending on participant’s choice. All participants saw 120 trials in a different semi-random order, with a maximum of three subsequent trials with the same outcome. There were three types of trials: (i) a Win face, associated with winning in 27 (90%), and with losing in three (10%) trials; (ii) a Lose face, associated with losing in 27 (90%), and with winning in three (10%) trials; and (iii) two Neutral faces, each shown in 30 trials and equally associated with winning, losing, or draws. At the end of the task, all participants were informed that they had won $4.70, which they immediately received in cash. The conditioning task was preceded by six practice trials, with the same procedure, but a different face identity.

The rating task followed the card-guessing game. Participants rated the valence and the intensity of emotional expressivity of the previously seen faces – shown in random order. The computer mouse was used to select a point on a horizontal 100-points Likert scale ranging from “Do not like it at all” on the left to “Like it a lot” on the right. Similarly, participants were asked on a separate screen to rate the intensity of the emotion displayed by the face from “No emotion at all” to “Very strong emotional expression.”

In the oddball task (Figure 1, bottom), which took place inside the MR scanner, participants watched 4-s long video clips showing dynamic facial expressions of anger or happiness by the previously seen Win and Lose faces, and by faces they had never seen before. The combination of facial identity and expression resulted in congruent trials (HappyWin, AngryLose) and incongruent trials (HappyLose, AngryWin). The task was presented in 3 runs of 61 trials, lasting 10.4 min each. Participants were allowed to rest between runs. Each run comprised 28 videos of the Win face (50% happy, 50% angry), 28 videos of the Lose face (50% happy, 50% angry), and 5 “oddballs,” i.e., faces never seen before. On oddball trials participants had to press, as quickly and accurately as possible, a button on an MRI-compatible button box using their right index finger. Each trial was composed of a central fixation cross (2–3 s, mean 2.5 s), the video clip (4 s), and a blank screen (2–5 s, mean 3.5 s).

### EMG Recording and Preprocessing

Bipolar EMG was acquired over the left ZM and corrugator supercilii (CS) muscles. Because data quality of the CS was poor only the ZM was included in the analyses. An EMG100C Biopac amplifier^2^ and EL510 MRI-compatible single-use electrodes were used, placed according to guidelines (Fridlund and Cacioppo, 1986). To provide a ground, an EL509 electrode was placed on the participant’s left index finger and attached to the negative pole of an EDA100C amplifier. Sampling rate was set to 10 kHz. Data were preprocessed offline, using Biopac’s Acqknowledge program, and self-made scripts in Matlab (version R2014b) partially using the EEGLAB toolbox (Delorme and Makeig, 2004). Data were filtered with a comb stop filter to remove frequencies around 20 Hz and all harmonics up to 5 kHz. Then a high-pass filter of 20 Hz and a low-pass filter of 500 Hz were applied. Data were downsampled to 500 Hz, segmented from 500 ms before to 4 s after stimulus onset, rectified, and smoothed with a 40 Hz filter. Separately for each fMRI run and participant, trials were excluded if their average amplitude in the baseline or post-stimulus-onset period exceeded by more than two SDs the average amplitude of all baselines or trials (for a similar procedure see Korb et al., 2015b). An average of 34 trials (*SD* = 2.8) per participant and condition (HappyWin, HappyLose, AngryWin, AngryLose) were available for statistical analyses. The average number of oddball trials was 11.5 (*SD* = 2.2).

### fMRI Recording and Preprocessing

Neuroimaging data were collected using a General Electric 3-T scanner with an eight-channel head coil. Functional images were acquired using a T2^*^-weighted gradient echo-planar imaging (EPI) sequence [40 sagittal slices/volume, 4 mm thickness, and 0 mm slice spacing; 64 × 64 matrix; 240-mm field of view; repetition time (TR) = 2000 ms, echo time (TE) = 24 ms, flip angle (FA) = 60°; voxel size = 3.75 × 3.75 × 4 mm; 311 whole-brain volumes per run]. To allow for the equilibration of the blood oxygenated level-dependent (BOLD) signal, the first six volumes (12 s) of each run were discarded. A high-resolution T1-weighted anatomical image was acquired in the beginning (T1-weighted inversion recovery fast gradient echo; axial scan with frequency direction A/P; 256 × 256 in-plane resolution; 256-mm field of view; FA = 12°; TI = 450; Receiver Bandwith = 31.25; 160 slices of 1 mm).

Blood oxygenated level-dependent data were processed with SPM12 (the Wellcome Department of Cognitive Neurology, London, United Kingdom^3^). To correct for head motion, the functional images were spatially realigned to the mean image of each run. Each participant’s anatomical images were co-registered to the mean EPI functional image, which was then normalized to the Montreal Neurological Institute (MNI) template. The parameters generated to normalize the mean EPI were used to normalize the functional images. Normalized images were resampled to 2 × 2 × 2 mm voxel size and spatially smoothed with a three-dimensional Gaussian filter (full-width at half maximum, 8 mm).

The preprocessed functional data were analyzed using a general linear model (GLM) with boxcar functions defined by the onsets and duration (4 s) of the videos and convolved with the canonical hemodynamic response function. Separate regressors were created for each condition (Anger-Win, Anger-Lose, Happy-Win, Happy-Lose) and temporal derivatives were included. An additional regressor was used to model all other events, including trials with rejected EMG data, oddball trials with button presses (hits), oddball trials without button presses (misses), and other button presses (false alarms). Finally, six motion correction parameters were included as regressors of no interest. A high-pass filter (128 s) was applied to remove low-frequency signal drifts, and serial correlations were corrected using an autoregressive AR(1) model.

Single-subject contrasts were taken to second level random effects analyses to find significant clusters across the subject sample, that can be generalized to the population. A region of interest (ROI) approach was used based on functional coordinates published in previous studies investigating the prefrontal modulation of spontaneous imitation of finger or hand movements (Brass et al., 2005, 2001). Additional whole-brain analyses were also performed and are provided in the Supplementary Material (see Supplementary Tables S1, S2).^4^ All activations are reported with a significance threshold of *p* < 0.001, uncorrected, and a cluster extent of *k* = 10. It should be noted that this thresholding only provides unquantified control of family-wise error (Poldrack et al., 2008; but see Lieberman and Cunningham, 2009), resulting in a high false-positive risk, and inferences based on them should be considered as preliminary (for stricter statistical thresholding with FWE correction, see right column in tables). Unthresholded whole-brain summaries for the main contrasts of interest are available at https://neurovault.org/collections/INQEMKIF/.

### Region of Interest (ROI) Analyses

A spherical ROI with a radius of 6 mm was created with the wfu_pickatlas toolbox (Maldjian et al., 2003) and placed around the coordinates reported by Brass et al. (2005; MNI: 1, 51, 12) to be the peak of a cluster activated during the inhibition of spontaneous imitation of finger movements. Beta-values were extracted and averaged over all voxels with the REX script (Duff et al., 2007), and averaged over sessions. Mean beta-values were analyzed in an rmANOVA with the factors Emotion (Happy, Angry) and Reward (Win, Lose).

To further rule out the possibility that specific experimental conditions were correlated with participants’ head movements, a similar method was used as in Johnstone et al. (2006). Specifically, for each participant and session the four regressors of interest (HappyWin, HappyLose, AngryWin, and AngryLose) were extracted from the SPM matrix and correlated with each of the six movement regressors. This resulted in 4 (regressors) × 6 (movement) × 3 (sessions) = 72 correlations per participant. We summed the number of significant correlations (*p* < 0.05, uncorrected) across movement regressors and sessions, and analyzed the resulting 35 (subjects) × 4 (conditions) matrix in a rmANOVA with the factors Emotion (Angry, Happy) and Reward (Win, Lose). No significant main or interaction effects were found (all *F* < 1.5, all *p* > 0.2), suggesting that conditions were not differentially correlated with movement in any specific direction (see Supplementary Figure S1). Head movement was thus comparable across all experimental conditions.

### Psychophysiological Interaction

Psychophysiological interaction analyses were computed to estimate condition-related changes in functional connectivity between brain areas (Friston et al., 1997; O’Reilly et al., 2012). In PPI analyses the time course of the functional activity in a specified seed region is used to model the activity in target brain regions. A model is created by multiplying the time course activity in the seed region (i.e., the physiological variable) with a binary comparison of task conditions (“1” and “−1”) (i.e., the psychological variable). Individual participants are modeled as additional conditions of no interest. Functional connectivity with the seed region is assumed if the brain activity in one or more target regions can be explained by the model. PPI analyses were used to estimate stimulus-related changes in functional connectivity with the mPFC (6 mm sphere centered at MNI: *x* 1, *y* 51, *z* 12). We tested the whole-brain functional connectivity in two contrasts of interest (Table 1).

**TABLE 1:** Group activations for PPI analyses.

**Region**	**Side**	**Coordinates (MNI)**			***z*-score**	**Cluster size**	***p*-FWE-corr**
		***x***	***y***	***z***			
**Incongruent > congruent**							
Supramarginal gyrus	Left	−60	−34	46	3.42	74	0.982
Posterior insula	Right	34	−24	22	3.19	67	0.985
Temporal white matter	Left	−24	−34	16	3.16	110	0.961
Lingual gyrus	Right	22	−50	2	3.13	60	0.987
Middle occipital gyrus	Right	32	−86	34	3.08	55	0.989
Parietal white matter	Left	−24	−20	44	3.00	33	0.995
Posterior insula	Left	−38	−4	18	2.96	40	0.993
Premotor cortex (precentral gyrus)	Right	22	−16	58	2.94	44	0.992
Lingual gyrus	Left	−14	−82	0	2.93	69	0.984
IFG (pars opercularis)	Right	30	8	38	2.93	66	0.985
Putamen	Right	26	18	12	2.90	26	0.996
Inferior temporal gyrus	Left	−54	−34	−16	2.90	14	0.998
Fusiform gyrus	Left	−28	−50	−2	2.89	30	0.996
Parahippocampal gyrus	Right	30	−28	−18	2.88	47	0.992
Caudate nucleus	Right	18	−2	22	2.86	40	0.993
Superior orbital gyrus	Right	24	40	−6	2.84	24	0.997
Anterior cingulum	Right	18	36	18	2.82	13	0.998
Supramarginal gyrus	Right	66	−30	38	2.79	17	0.998
Parahippocampal gyrus	Left	−30	−28	−14	2.75	20	0.997
Midcingulate cortex	Left	−10	−20	48	2.75	17	0.998
Cuneus	Right	8	−92	22	2.71	18	0.998
**Congruent > incongruent**							
Parahippocampal gyrus	Left	−10	−14	−22	3.55	31	0.996

*Clusters of 10 or more contiguous voxels whose global maxima meet a threshold of p < 0.001 uncorrected are reported. FWE-corrected p-values (cluster-level) are also reported. Regions of activation are listed with best estimates of anatomical location.*

## Results

### Ratings

Ratings of liking, provided after the conditioning experiment, were analyzed in a one-way rmANOVA with the levels Win, Lose, No change. A significant effect of Reward [*F*(2,80) = 28.52, *p* < 0.001, $\eta_{p}^{2}$ = 0.451) was found. *Post hoc t*-tests revealed that faces associated with losing money were rated significantly lower than those associated with winning and with no change (all *t* > 5.7, all *p* < 0.001). Ratings for Win and No change faces did not differ [*t*(34) = 0.9, *p* = 0.34]. An identical ANOVA carried out on the ratings of intensity did not result in a significant effect of condition [*F*(2,80) = 0.4, *p* < 0.67, $\eta_{p}^{2}$ = 0.01).

### EMG

Electromyography of the ZM was analyzed in an Emotion (Happy, Angry) × Reward (Win, Lose) × Time (seconds 1–4 from stimulus onset) rmANOVA. This resulted in significant effects of Reward [*F*(1,38) = 4.8, *p* = 0.035, $\eta_{p}^{2}$ = 0.11], Time [*F*(3,114) = 4.9, *p* = 0.015, $\eta_{p}^{2}$ = 0.11], Emotion × Reward [*F*(1,38) = 4.8, p = 0.034, $\eta_{p}^{2}$ = 0.11], Emotion × Time [*F*(3,114) = 3.3, *p* = 0.050, $\eta_{p}^{2}$ = 0.08], and Emotion × Reward × Time [*F*(3,114) = 3.2, *p* = 0.036, $\eta_{p}^{2}$ = 0.08]. The main effect of Emotion was not significant (*F* < 1.9, *p* > 0.18). As the three-way interaction was significant, we proceeded by running separate analyses for each emotion.

**FIGURE 2:**
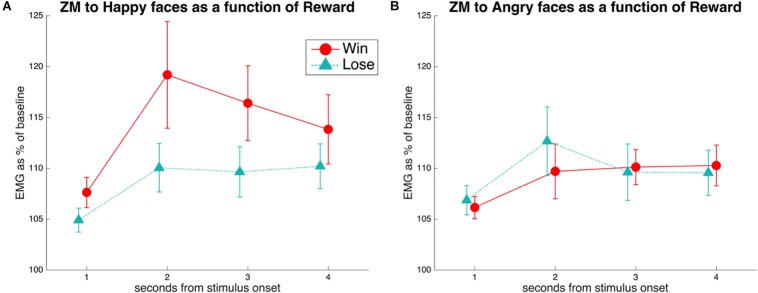
**(A)** EMG of the ZM in response to happiness in faces associated with winning or losing money. A main effect of Reward was found, due to reduced mimicry of smiles when the face was associated with losing compared to winning money. This difference was significant at Times 1–3, as shown by pairwise comparisons. **(B)** EMG of the ZM in response to anger in faces associated with winning or losing money. No significant differences were found.

A rmANOVA on the EMG to happy stimuli, with the factors Reward (Win, Lose) and Time (seconds 1–4 from stimulus onset), resulted (Figure 2A) in a significant main effect of Reward [*F*(1,38) = 7.8, *p* = 0.008, $\eta_{p}^{2}$ = 0.17], with greater ZM activation to Win faces (*M* = 113.2, *SE* = 2.8) than to Lose faces (*M* = 108.1, *SE* = 1.7). The main effect of Time was also significant [*F*(3,114) = 6.8, *p* = 0.004, $\eta_{p}^{2}$ = 0.15], with lowest values in the first second after stimulus onset (*M* = 106.1, *SE* = 1.1), and higher values in the three following seconds (respectively, *M* = 113.6, 112.0, 110.8, *SE* = 3.3, 2.5, 2.3). The Reward × Time interaction was not significant [*F*(3,114) = 1.9, *p* = 0.13, $\eta_{p}^{2}$ = 0.05]. Exploratory *t*-tests suggested a difference in ZM activation to happy stimuli for Win vs. Lose in the first to third second [respectively, *t*(38) values = 3.3, 2.8, 2.2; *p*-values = 0.002, 0.008, 0.034], but not in the last second after stimulus onset [*t*(38) = 1.31, *p* = 0.196].

A similar rmANOVA of the ZM in response to angry faces (Figure 2B) revealed a barely significant Time × Reward interaction [*F*(3,114) = 2.78, *p* = 0.05, $\eta_{p}^{2}$ = 0.068]. However, pairwise comparisons between reward levels at every time point were all not significant (all *t* < 1.7, all *p* > 0.1).

### fMRI

We hypothesized that the mPFC would be more activated for trials involving an incongruent combination of expression valence and conditioned association (HappyLose and AngryWin) than for trials with congruent stimuli (HappyWin and AngryLose). The reason is that in such cases spontaneous facial mimicry should be inhibited in a top-down manner.

**FIGURE 3:**
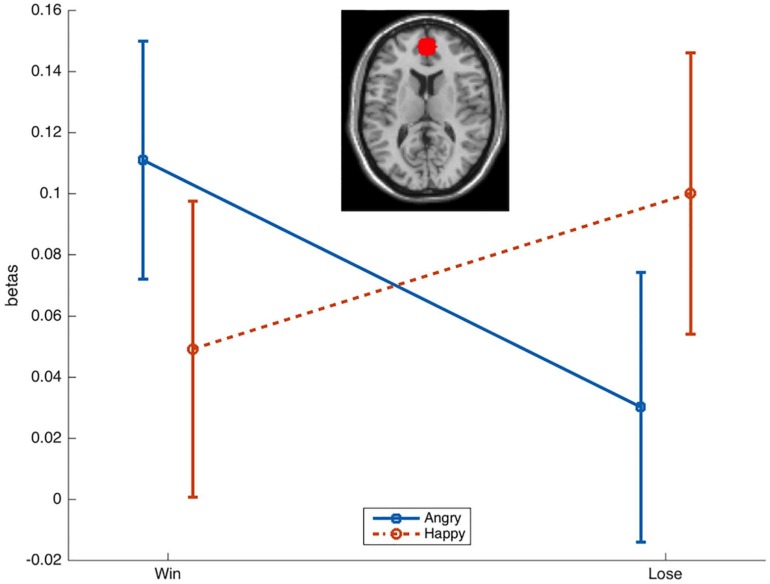
Mean (*SE*) beta-values from the ROI in the mPFC. A significant Emotion × Reward interaction was characterized by greater activation to incongruent (AngryWin, HappyLose) compared to congruent (AngryLose, HappyWin) trials.

Activation in the *a priori* defined ROI was compared across conditions with rmANOVAs with the factors Emotion (Happy, Angry) and Reward (Win, Lose). This resulted (Figure 3) in a significant Emotion × Reward interaction [*F*(1,34) = 4.7, *p* = 0.036, $\eta_{p}^{2}$ = 0.12]. *T*-tests only resulted in a near-to-significant difference [*t*(34) = 1.9, *p* = 0.07] between AngryWin faces (*M* = 0.11, *SE* = 0.04) and AngryLose faces (*M* = 0.3, *SE* = 0.04). Other comparisons were not significant (all *t* < 1.5, all *p* > 0.17).

### Psychophysiological Interaction

**FIGURE 4:**
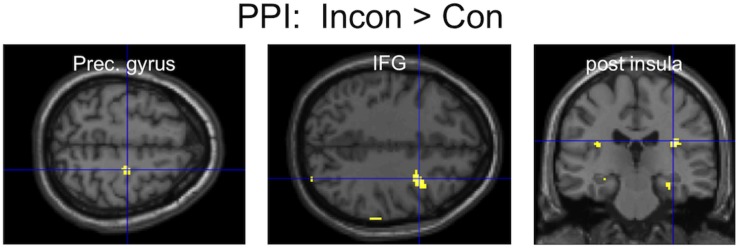
PPI results: three clusters of interest showing increased functional connectivity with the mPFC for the contrast Incongruent > Congruent.

An area of the mPFC (MNI: 1, 51,12), previously reported to be involved in the inhibition of finger movement imitation (Brass et al., 2005), showed greater functional connectivity during Incongruent than Congruent trials with several brain areas, most notably (Figure 4) including the bilateral posterior insula and the right motor cortex (precentral gyrus) and inferior frontal gyrus (IFG). The opposite contrast only showed greater connectivity with the parahippocampal gyrus (Table 1).

## Discussion

The reported fMRI and facial EMG study investigated, for the first time, the neural correlates of the inhibition of spontaneous facial mimicry.

As expected, facial mimicry of happiness was reduced for disliked faces (i.e., faces associated with losing money), compared to liked faces (i.e., faces associated with winning money). This replicates previous findings in which participants were conditioned to associate specific facial identities with positive or negative monetary outcomes (Hofman et al., 2012; Sims et al., 2012). Of particular interest is the comparison with the study by Sims et al. (2012), from which we borrowed the stimuli and most of the procedure (excluding the 60% faces). Despite the difficulties linked to recording low-amplitude facial EMG in the fMRI scanner, the ZM pattern in response to HappyWin and HappyLose is very similar across the two studies (compare Figure 2A here with their Figure 2). If anything, the effect size of the main effect of Reward was greater in our study ($\eta_{p}^{2}$ = 0.16) than in that by Sims and colleagues ($\eta_{p}^{2}$ = 0.09) – although the data processing and analysis procedures are not entirely comparable.

The replication of previously published reports that the amplitude of facial mimicry is modulated by the observer’s liking/disliking of the sender is relevant for several reasons. First, it is significant in light of recent failures to replicate high-profile findings, especially in the literature on embodied cognition (Wagenmakers et al., 2016; but see Noah et al., 2018), which have thrown light upon the need to replicate experimental results in psychology. Second, the findings are significant because we replicate past effects even with the additional difficulty of recording faint changes in facial muscle contraction, as they are typical for facial mimicry, during MRI. To the best of our knowledge, very few published studies have successfully simultaneously recorded facial EMG and BOLD data with fMRI (Heller et al., 2011; Likowski et al., 2012; Rymarczyk et al., 2018). Thus, the present report adds to the growing literature demonstrating the technical feasibility of monitoring facial EMG during fMRI scanning. Usage of dynamic face stimuli, as opposed to static pictures, is advisable in such EMG–fMRI combined experiments, because they are known to elicit stronger facial mimicry (Sato et al., 2008).

It is important to point out that, although we employed the same task and stimuli as Sims et al. (2012), and also replicate their EMG findings, our interpretation of the change in smile mimicry between HappyWin and HappyLose expressions differs. Specifically, Sims and colleagues interpret the difference in smile mimicry, between faces associated with winning and losing money, as indicating that the reward value of the face leads to *increased* mimicry. In their view, reward *augments* facial mimicry of smiles. In contrast, in our view, incongruence between the valence of a facial expression (Happy, Angry) and the memory (Win, Loss) attached to it results in the inhibition of facial mimicry. Accordingly, we interpret the smaller ZM activation for HappyLose compared to HappyWin faces as *inhibiting* spontaneous facial mimicry. This difference in focus is not due to a fundamental disparity in belief about the mechanisms underlying facial mimicry and its modulation, but instead to the lack of a neutral association condition in this paradigm. Previous studies (Likowski et al., 2011; Hofman et al., 2012; Seibt et al., 2013) have indeed shown that facial mimicry can be modulated by both positive associations (e.g., fair monetary offers, cooperation priming) and negative associations (e.g., unfair monetary offers, competition priming). When compared with a neutral condition, effects of negative associations on facial mimicry may be even slightly more frequent (Likowski et al., 2011; Hofman et al., 2012; Seibt et al., 2013), which supports our interpretation of the difference in ZM activation to HappyWin and HappyLose faces as the result of reduced facial mimicry to HappyLose faces. The effects of reward and punishment on facial mimicry are however not mutually exclusive, and in fact we have interpreted the results based on the congruence/incongruence between the valence of the expression and the valence of the monetary contingency associated with it.

Functional magnetic resonance imaging results show that the mPFC became more active during incongruent trials, (i.e., happy faces associated with losing and angry faces associated with winning, than during congruent trials). This was expected in light of brain imaging studies reporting that the mPFC is part of a network underlying the capacity to differentiate between one’s own and others’ movements, and to inhibit the imitation of perceived finger/hand movements (Brass et al., 2001, 2005, 2009; Brass and Haggard, 2007; Kühn et al., 2009; Wang et al., 2011; Wang and Hamilton, 2012). The finding is also consistent with the fact that patients who suffered lesions in prefrontal areas of the brain have difficulties to inhibit prepotent response tendencies, and show an accrued tendency to imitate other people’s actions (Lhermitte et al., 1986; De Renzi et al., 1996; Brass et al., 2003). In a slightly more dorsal area, cortical thickness of the mPFC was found to correlate with the tendency to suppress emotional expressions in everyday life, as measured by questionnaire (Kühn et al., 2011). Areas of the mPFC were also shown to underlie other aspects of social cognition, including the monitoring of eye gaze, person perception, and mentalizing (Amodio and Frith, 2006). The results reported here suggest that the mPFC is also involved in the top-down inhibition of spontaneous facial mimicry. In line with this, the mPFC has been proposed to be a key node in a distributed brain network underlying the processing of and responding to facial expressions in their context (Kraaijenvanger et al., 2017). Yet, the brain circuitry supporting the inhibition of facial mimicry could still differ from that supporting the inhibition of finger movements. Therefore, further research, including with other neuroscientific methods (e.g., lesion studies, TMS), is necessary. For example, it remains unclear if the tendency of prefrontal patients to over-imitate movements and actions of the limbs (De Renzi et al., 1996; Brass et al., 2003) also extends to facial expressions (McDonald et al., 2010).

Incongruent trials also resulted in increased functional connectivity between the mPFC, right motor cortices (precentral gyrus and IFG), and limbic areas (insula), as suggested by PPI analyses. Although results were only apparent at more liberal statistical thresholding, and PPI might not be the best tool to reveal the *direction* of functional connectivity, it can be speculated that during incongruent trials the mPFC inhibits motor and limbic areas, which are involved in the generation of facial mimicry and the judgment of facial expressions, as suggested by recent TMS studies (Korb et al., 2015b; Paracampo et al., 2016), as well as brain models (Kraaijenvanger et al., 2017). Indeed, right somatomotor areas appear to be crucially involved in the production of facial mimicry and in the integration of facial feedback for the recognition of emotional facial expressions (Adolphs et al., 2000; Pourtois et al., 2004; Pitcher et al., 2008; Keysers et al., 2010; Korb et al., 2015b; Paracampo et al., 2016). The IFG was repeatedly shown to underlie the imitation of gestures and finger movements (Heyes, 2001). Interestingly, in a task requiring the inhibition of incongruent hand gestures a close-by area of the mPFC was also found to inhibit the IFG, in addition to the STS, as shown by dynamic causal modeling (Wang et al., 2011). The insula has also been associated with the readout of facial feedback, as well as interoception in general (Adolphs et al., 2000; Critchley et al., 2004). Therefore, one can speculate that the increased connectivity between the mPFC and the bilateral (however posterior instead of anterior) insula reflects the prefrontal inhibition of emotional responses to stimuli with incongruent valence of expression and contingency.

The finding of increased activation of the mPFC during incongruent trials, and of its likely inhibition of somatomotor and interoceptive areas, is relevant in light of a long-lasting discussion in social psychology/neuroscience. In fact, based on numerous reports of the modulation of facial mimicry by context and other factors (for recent reviews see Seibt et al., 2015; Kraaijenvanger et al., 2017), opponents of the “matched motor hypothesis” have often dismissed the view that facial mimicry consists of a spontaneous and fast (reflex-like), motor response (Hess and Fischer, 2014), which takes place when encountering other people’s emotional expressions. Here, however, we reveal that activation of an area of the mPFC, previously shown to be crucially involved in the inhibition of the tendency to imitate finger movements, also accompanies the inhibition of smile mimicry of disliked faces. This finding suggests that facial mimicry does indeed arise quickly and spontaneously (be this reaction inborn or automaticed through early associative learning and repeated performance, see for example Heyes, 2001), but can be inhibited or otherwise modulated in a top-down manner through the activity of regulatory prefrontal cortices. Similarly, the STORM model proposed that the mPFC connects to and controls areas of the MNS, from where imitation arises (Wang and Hamilton, 2012).

It is important to point out that opponents of the “matched motor hypothesis” have argued that evidence of a situation-dependent modulation of facial mimicry, and other forms of imitation, necessarily rules out the hypothesis that these behaviors arise in a quick “reflex-like” way. We, on the other hand, are less categorical, and would like to remind the field that proof of mimicry modulation cannot, by any means, be considered sufficient evidence against the quickness and spontaneity of a behavior or physiological reaction. After all, even spinal reflexes can be inhibited in a, literally, top-down fashion. In line with definitions of automaticity proposed by Kahneman and Treisman (1984) and Kornblum et al. (1990), we would like to argue that facial mimicry has at least the qualities of partial automaticity.

A limitation of this study is the lack of a neutral condition, in which facial expressions of familiar faces are shown that are not associated with winning or with losing. Inclusion of this neutral condition would have provided a baseline, to which the changes in facial mimicry for congruent and incongruent trials could have been compared (e.g., see Hofman et al., 2012). It should also be noted that the here reported fMRI results were based on relatively liberal statistical thresholds, which increase the false-positive risk (for stricter statistical thresholding with FWE correction, see right column in tables), and inferences based on them should be considered as preliminary evidence awaiting replication in a bigger sample. However, the pattern of BOLD responses in the mPFC shown here corresponds to predictions based on an extended literature investigating brain responses during the observation of motor actions and their inhibition (finger tapping incongruent to one’s own). This work also adds to the burgeoning literature of concurrent EMG and fMRI of facial mimicry.

## Conclusion

In conclusion, the view of disliked compared to liked smiling faces resulted in reduced facial mimicry, as shown with EMG. Cortical activity and connectivity, as measured with fMRI and PPI, suggested that the reduction in facial mimicry is caused by inhibition of somatomotor and insular cortices originating from the mPFC. The same medial prefrontal area thus allows the inhibition of imitative finger/hand movements, and of facial mimicry.

## Data Availability

The datasets generated for this study are available on request to the corresponding author.

## Ethics Statement

All participants signed the informed consent. The study was approved by the Health Sciences IRB of the University of Wisconsin–Madison (FWA00005399).

## Author Contributions

SK and PN designed the experiment. SK and RG acquired and analyzed the data. SK, RG, RD, and PN wrote the manuscript.

## Conflict of Interest Statement

The authors declare that the research was conducted in the absence of any commercial or financial relationships that could be construed as a potential conflict of interest.

## Footnotes

1 www.jkp.com/mindreading

2 www.biopac.com

3 www.fil.ion.ucl.ac.uk

4 We also carried out analyses with the EMG of the ZM (averaged over the length of the video) as a parametric modulator of the BOLD at the first level analyses. At the second level, this did not result in greater BOLD for the contrasts of interest (e.g., HappyWin > HappyLose) at the threshold of *p* < 0.001. This could be due to the EMG being fairly noisy when recorded in the scanner, given interference of the gradient noise and cardiac-related noise. We therefore opted for the simpler analyses, here reported.
