# Supplementary material for: Increased Medial Prefrontal Cortex and Decreased Zygomaticus Activation in Response to Disliked Smiles Suggest Top-Down Inhibition of Facial Mimicry
Source: Front Psychol. 2019 Jul 26;10:1715. doi: 10.3389/fpsyg.2019.01715 (PMC6677088; doi:10.3389/fpsyg.2019.01715)
Supplement: Supplementary file 1 [file Data_Sheet_1.PDF]

Supplementary material

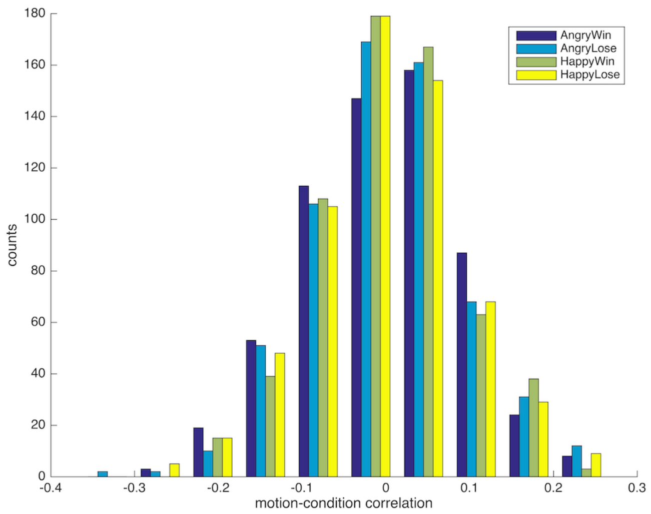

Figure S1: histogram of the correlation coefficients resulting from correlating the regressors of interest with the 6 movement regressors. Most coefficients were small, and the number of significant correlations did not differ across conditions.

Table S1
Group activations for Emotion contrasts

Region	Side	Coordinates (MNI)			*z* score	Cluster size	*p* FWE-corr
		x	*y*	*z*			
**Angry > Happy**							
Middle Frontal Gyrus	Left	-40	26	40	4.81	922	.005
Insula Lobe	Left	-32	12	14	4.35	114	.486
Superior Frontal Gyrus	Right	24	50	22	4.35	342	.107
Middle Frontal Gyrus	Right	38	32	40	4.30	276	.163
Linual Gyrus	Left	-4	-70	4	4.17	199	.272
Middle Temporal Gyrus	Left	-54	-72	4	4.16	1457	.000
Middle Cingulum	Right	14	16	30	4.1	76	.625
Insula	Right	34	-10	26	3.99	81	.605
Superior Frontal Gyrus	Right	18	18	60	3.95	145	.394
SupraMarginal Gyrus	Right	56	-44	30	3.94	474	.048
Hippocampus	Right	40	-12	-20	3.84	77	.621
Inferior Temporal Gyrus	Right	52	-50	-22	3.83	65	.671
Anterior Cingulum	Left	-10	24	22	3.82	331	.114
Middle Frontal Gyrus	Left	-24	38	22	3.75	129	.439
Middle Cingulum	Left	-4	-16	32	3.70	129	.439
Inferior Temporal Gyrus	Right	48	-30	-18	3.52	63	.679
Insula	Right	36	6	-2	3.49	39	.786
IFG (p. Triangularis)	Right	48	36	20	3.44	50	.736
Posterior Cingulum	Left	-8	-40	22	3.44	32	.817
Hippocampus	Right	34	-38	-4	3.41	12	.908
Rolandic Operculum	Left	-56	2	10	3.39	187	.295
Middle Cingulum	Right	12	0	36	3.36	15	.894
IFG (p. Opercularis)	Right	62	14	12	3.36	24	.854
Middle Frontal Gyrus	Left	-40	4	56	3.32	40	.781
Prefrontal white matter	Right	24	-4	44	3.27	15	.894
IFG (p. Triangularis)	Left	-36	24	12	3.25	11	.912
Thalamus	Right	14	-30	22	3.24	11	.912
IFG (p. Orbitalis)	Right	52	22	2	3.19	10	.917
Inferior Parietal Lobule	Left	-54	-42	42	3.17	10	.917
**Happy > Angry**							
Superior Occipital Gyrus	Left	-14	-100	10	4.38	496	.042
Calcarine Gyrus	Right	16	-96	10	4.18	129	.439
Superior Occipital Gyrus	Right	18	-98	24	3.52	22	.863

Note. Clusters of 10 or more contiguous voxels whose global maxima meet a threshold of $p<.001$ uncorrected, are reported. FWE-corrected p values (cluster-level) are also reported. Regions of activation are listed with best estimates of anatomical location based on the Tailarach Daemon (Lancaster et al., 2000) and on the Anatomy Toolbox (Eickhoff et al., 2005).

Table S2
Group activations for Reward contrasts

Region	Side	Coordinates (MNI)			*z* score	Cluster size	*p* FWE-corr
		x	*y*	*z*			
**Win > Lose**							
Hippocampus	Right	26	-34	14	3.72	37	.795
Middle Frontal Gyrus	Right	30	56	32	3.41	13	.903
**Lose > Win**							
No suprathreshold clusters							

Note. Clusters of 10 or more contiguous voxels whose global maxima meet a threshold of $p<.001$ uncorrected, are reported. FWE-corrected *p* values (cluster-level) are also reported. Regions of activation are listed with best estimates of anatomical location based on the Tailarach Daemon (Lancaster et al., 2000) and on the Anatomy Toolbox (Eickhoff et al., 2005).
